# Significant Association Between Variant in *SGCD* and Age-Related Macular Degeneration

**DOI:** 10.3390/genes9100467

**Published:** 2018-09-25

**Authors:** Andric Christopher Perez-Ortiz, Alexandra Luna-Angulo, Juan Carlos Zenteno, Alvaro Rendon, Liliana Guadalupe Cortes-Ballinas, David Jimenez-Collado, Bani Antonio-Aguirre, Martha Janneth Peralta-Ildefonso, Israel Ramírez, Stefany Jacob-Kuttothara, Francisco Javier Estrada-Mena

**Affiliations:** 1 Laboratorio de Biología Molecular, Escuela de Medicina, Universidad Panamericana, Donatello 59 Insurgentes Mixcoac Benito Juárez 03920 Ciudad de México, Mexico; lunangulo@gmail.com (A.L.-A.); lilixar@gmail.com (L.G.C.-B.); djimcoll@gmail.com (D.J.-C.); bani.aantonio@gmail.com (B.A.-A.); martha.janneth2830@gmail.com (M.J.P.-I.); israel.ramirez04@gmail.com (I.R.); skuttothara@gmail.com (S.J.-K.); 2 Laboratory of Epidemiology and Public Health (LEPH), Yale University School of Public Health, New Haven, CT 06510, USA; andric@aya.yale.edu; 3 Departamento de Neurociencias, Instituto Nacional De Rehabilitación, Calzada México-Xochimilco 289, Arenal Tepepan, 14389 Ciudad de México, Mexico; 4 Genetics Department, Research Unit, Institute of Ophthalmology Conde de Valenciana Foundation Department of Biochemistry, Faculty of Medicine, Universidad Nacional Autónoma de México, 06080 Ciudad de México, Mexico; jczenteno@institutodeoftalmologia.org; 5 Institut De La Vision, Sorbonne Universites, F-75012 Paris, France; alvaro.rendon@inserm.fr; 6 Facultad de Química, Universidad Nacional Autónoma de México, Coyoacán, Ciudad Universitaria, 04510 Ciudad de México, Mexico

**Keywords:** *SGCD*, delta-sarcoglycan, candidate-gene approach, AMD

## Abstract

*CFH* and *HTRA1* genes are traditional markers of increased risk of age-related macular degeneration (AMD) across populations. Recent findings suggest that additional genes—for instance, in the dystrophin-associated protein complex—might be promising markers for AMD. Here, we performed a case-control study to assess the effect of *SGCD* single nucleotide polymorphisms (SNPs), a member of this protein family, on AMD diagnosis and phenotype. We performed a case-control study of an under-studied population from Hispanics in Mexico City, with 134 cases with 134 unpaired controls. Cases were 60 years or older (Clinical Age-Related Maculopathy Staging (CARMS) grade 4–5, as assessed by experienced ophthalmologists following the American Association of Ophthalmology (AAO) guidelines), without other retinal disease or history of vitreous-retinal surgery. Controls were outpatients aged 60 years or older, with no drusen or retinal pigment epithelium (RPE) changes on a fundus exam and a negative family history of AMD. We examined SNPs in the *SGCD* gene (rs931798, rs140617, rs140616, and rs970476) by sequencing and real-time PCR. Genotyping quality checks and univariate analyses were performed with PLINK v1.90b3.42. Furthermore, logistic regression models were done in SAS v.9.4 and haplotype configurations in R v.3.3.1. After adjusting for clinical covariates, the G/A genotype of the SGCD gene (rs931798) significantly increases the odds of being diagnosed with AMD in 81% of cases (1.81; 95% CI 1.06–3.14; *p* = 0.031), especially the geographic atrophy phenotype (1.82; 95% CI 1.03–3.21; *p* = 0.038) compared to the G/G homozygous genotype. Moreover, the GATT haplotype in this gene (rs931798, rs140617, rs140616, and rs970476) is associated with lower odds of AMD (adjusted odds ratio (OR) 0.13; 95% CI 0.02–0.91; *p* = 0.041). *SGCD* is a promising gene for AMD research. Further corroboration in other populations is warranted, especially among other Hispanic ethnicities.

## 1. Introduction

Age-related macular degeneration (AMD) is the leading cause of blindness in individuals over 60 years old in developed countries [1], and the third leading cause worldwide [2]. This condition accounts for ~14% of all legal blindness [1,3]. AMD is a complex, polygenic, and multifactorial disease characterized by progressive photoreceptor degeneration and cell death [4]. During the early stage of the disease, most patients have no noticeable decline in visual acuity [5], even though drusen deposits and pigmentary changes are already observed by fundoscopy [6]. Over a five-year period from the onset of the disease, up to 5% of asymptomatic patients will develop one or a combination of two advanced forms of AMD: (1) choroidal neovascularization (CNV, exudative, or wet AMD) or (2) geographic atrophy (GA) of the retinal pigment epithelium (nonexudative or dry AMD) [7]. Such progression is not homogeneous across race/ethnicities, clearly indicating an effect modification in the development and progression of the AMD, primarily due to a genetic component. [8] Both GA and CNV are more prevalent among cases of European descent (~12.3 to 30% estimated prevalence), closely followed by Hispanics, the second largest affected race/ethnicity (~10.4%), then Africans (7.5%) and Asians (7.4%) [1]. However, among Hispanics, especially those from Latin America, these subtle differences in disease prevalence and rate of progression are understudied. It is clear that these advanced forms have a complex genetic background currently not thoroughly characterized, arguing for the need of study of new genetic markers in non-traditional loci.

Even though the AMD phenotype varies, both forms GA and CNV share several susceptibility genes, including two major loci in complement factor H (*CFH*) [9] and the age-related maculopathy susceptibility 2 (*ARMS2*)/HtrA serine peptidase 1 (*HTRA1*) [10]. Additionally, through genome-wide association (GWAS) and case-control studies, multiple genes in the complement pathway (*C2, CFB, C3, CFL,* and *VNT*) [11], lipids and high-density lipoprotein (HDL) cholesterol metabolism (*LIPC, CETP,* and *APOE*) [12], oxidative stress, angiogenesis (*VEFGA*), and other candidate genes in several biological pathways (*TRL3* and *TRL4*) have been identified [13]. The importance of the identification of these genetic variants is that they might influence the onset, progression, and possibly the response to treatment [7]. Currently, there are a total of 52 independently associated common and rare variants distributed across 34 loci. Since AMD is a multifactorial disease, many more loci might not be entirely elucidated yet [14]. Of importance, recently, an epistatic module describing the interaction of multiple loci for AMD proposed the *SGCD* gene (an unexpected result) as a possible bridge between the aging process and the immune dysregulation in AMD [15]. Tang et al. evidenced in silico a significant interaction between *SGCD* and the *SCAPER* gene that correlated with the *MASP1* and *MASP2* genes, which activate the complement pathway [15]. Additionally, Lin al., in a secondary prioritized subset analysis of an AMD GWAS, identified several in-silico single nucleotide polymorphisms (SNPs) in the *SGCD* gene significantly associated with AMD [16]. In silico analyses allow reviewing thousands of genes, and have proven to be useful in guiding clinical research. Bioinformatics analyses so far have elucidated both findings, but no study has assessed these relationships in vivo.

The *SGCD* gene located on chromosome 5q33.2-q33.3 encodes for δ-sarcoglycan (δ-SG), an integral membrane protein part of the sarcoglycan–sarcospan complex. δ-SG also is an essential component of the dystrophin-associated protein complex (DAPC). Sarcoglycans (SGs). as with other DAPCs, were first described in muscular tissues, where they promote muscle membrane stability. Together with the dystrophin–glycoprotein complex, SGs also connect the extracellular matrix with the cytoskeleton and regulate signal transduction [17]. Molecular alterations in the *SGCD* gene have been linked to limb–girdle muscular dystrophy type 2 (LGMD2F) and dilated cardiomyopathy type 1 L [18]. LGMD2F patients have visual disturbances not fully elucidated; such observational evidence underscores a potential role of SGs in retinal function [19].

In the murine retina, SGs are partially dystrophin independent [20]. δ-SG is predominantly expressed in the outer retina, near the Müller glial cell fibers and the outer limiting membrane [20]. The correlation between the location of δ-SG expression and the higher metabolic regions of the retina might be indicative its function, being the degradation of extracellular matrix through different enzymes, such as matrix metalloproteinases, cell cycle regulation, and DNA repair [15]. Some *SGCD* polymorphisms have also been implicated in drusen formation [15]. Furthermore, in a manuscript in preparation we have evidenced that the deficient SGCD-null mice exhibited signs of retinal degeneration and frailty, highlighting the pivotal role of *SGCD* for the normal retinal function [21]. To date, there is limited or no information evaluating the relationship between *SGCD* polymorphisms, those reported in silico, and the advanced forms of AMD. Hence, we aim to assess the effect of *SGCD* polymorphisms on AMD diagnosis and phenotype, using a candidate-gene approach among Hispanics from Mexico.

## 2. Materials and Methods

We performed a hospital-based case-control study of 134 age-related macular degeneration cases with 134 unpaired controls. All participants were recruited at the Institute of Ophthalmology (Conde de Valenciana) in Mexico City, and agreed to participate by signing informed consents. Hispanic and Mexican heritage was assessed at recruitment by self-identification and supported by the family history of last names and by two grandparents in two generations being born in Mexico City. Our sample size was calculated in R version 3.5.1 [22] with the “GeneticsDesign” package [22], by taking 80% power to detect a significant difference in odds ≥ 1.50, considering the allelic frequency of our SNPs. Moreover, before recruitment, we corroborated these calculations in PLINK v1.90b3.42 [23] and SAS v9.4. [24]. A total of 134 incident cases were assessed, and their status was assigned by experienced blinded ophthalmologists who stratified AMD phenotypes following the American Association of Ophthalmology (AAO) clinical guidelines [25]. Controls were taken randomly from a pool at our clinic and were those without any evidence of advanced AMD at baseline but who could develop it. Our full case and control definition is displayed in Supplementary Table S1. Briefly, all of our cases and controls were 50 years old or older, and there was no maximum age cap. We excluded cases with ophthalmological diseases that limited a thorough retinal examination by fundoscopy (e.g., cataracts), as well as those who withdrew their informed consent. To ascertain exposure, we recorded demographic characteristics and explicitly known risk factors for AMD, from electronic medical records such as age, sex, smoking history and other clinical comorbidities (hypertension and diabetes). For our genotype data, exposed cases were those carriers of the heterozygous or less frequent homozygous alleles (see below, Statistical Approach). All of our assays were done in duplicate by blinded experienced laboratory technicians. We genotyped four SNPs in the *SGCD* gene significantly associated with AMD in the bioinformatic analyses of Lin et al. [16]. Each of these was developed by two methods. First, for the SNP rs970476, we performed allelic discrimination assays with TaqMan probes (Thermo Fisher Scientific Inc., Wilmington, DE, USA) on real-time (RT) PCR by duplicate. The rest (rs931798, rs140617, and rs140616) were close enough (less than 300 base pairs across) to be genotyped by automated sequencing (see below, DNA Sequencing). The Institutional Review Boards approved this study at the Institute of Ophthalmology Conde de Valenciana (clinical site) and Universidad Panamericana (genotyping site) (CEI-2014-/02/01). Our procedures and data collection followed the tenets of the Declaration of Helsinki, and all data was handled as directed by Health Insurance Portability and Accountability Act (HIPAA).

To allocate exposure in our cases and controls, we began by extracting genomic DNA from peripheral blood samples that were collected in ethylenediaminetetraacetic acid (EDTA) tubes at baseline. We then immediately processed them using the PureGene DNA purification whole blood kit (QIAGEN, Germantown, MD, USA) following the manufacturers’ specifications. DNA concentrations and purity were quantified using a Multiskan™ GO Spectrophotometer (Thermo Fisher Scientific Inc., Wilmington, DE, USA). To evaluate DNA integrity, we performed a 0.8% agarose (Thermo Fisher Scientific Inc.) gel stained with GelRed (Biotium Corporate Headquarters Inc., Landing Pkwy, Fremont, CA, USA).

We genotyped the rs970476 of the *SGCD* gene with TaqMan SNP assay probes (Applied Biosystems, Foster City, CA, USA). We allocated a genotype to each case, following conventional methods of melting curve analyses for real-time PCR with the PikoReal Real-Time PCR System (Thermo Fisher Scientific Inc.). To ensure reproducibility and precision of our data, all assays were done in duplicate by blinded experienced laboratory technicians.

We mapped the three SNPs of interest to us, which were those reported by Lin et al. [16], in the second intron of *SGCD* gene (rs931798, rs140617, and rs140616), using the Ensembl genome browser 89 (http://www.ensembl.org/index.html). Since they are located less than 300 base pairs (bp) apart, we designed the following primers with Primer3 v0.4.0 (http://bioinfo.ut.ee/primer3-0.4.0/primer3/) for automated sequencing, 5′ TGGCCCTTGGCTATCTCTTC 3′ and 5′ TCATGCTCATCCTAGGGTCCA 3′, under these parameters: 283 bp as the length of a theoretical fragment, 50% CG content, and an alignment temperature of 54 °C. These primers were synthesized at the Institute of Cellular Physiology (Universidad Nacional Autónoma de México). For the PCR reaction, we used the AmpliTaq Gold DNA Polymerase (Applied Biosystems) under an alignment temperature of 54 °C using a Gradient Palm-Cycler™ (Corbett Life Science, Sydney, Australia). To verify DNA amplification and integrity, we ran 2.0% agarose gels (Thermo Fisher Scientific Inc.) stained with GelRed (Biotium Corporate Headquarters Inc.). Amplicons were compared to an O’RangeRuler 100 bp DNA Ladder (Thermo Fisher Scientific Inc.). Our primers, per design, needed to be within 300 bp. To eliminate oligonucleotides and salt remnants, we purified the PCR products with DNA Clean and Concentrator-5 (Zymo Research, Irvine, CA, United States). After cleaning up, all samples were likewise verified in a 2.0% agarose gel stained with GelRed, and their concentration was measured using a MassRuler Expres LR reverse DNA ladder (Thermo Fisher Scientific Inc.). Next, purified amplicons were marked with BigDye Terminator V3.1 Cycle Sequencing Kit (Applied Biosystems) and a PCR reaction was held in a Gradient Palm-Cycler (Corbett Life Science) following manufacturers’ specifications. The marked DNA was purified with the ZR DNA Sequencing Clean-up Kit (Zymo Research). A total of 30 ng was used to perform a capillary electrophoresis and sequencing at 50 °C with the ABI PRISM 310 Genetic Analyzer (Thermo Fisher Scientific Inc.). Electropherograms were analyzed conventionally with FinchTv v1.4. (Geospiza, Inc., Seattle, WA, United States; http://www.geospiza.com).

We began by describing the full sample, and then performed stratified analyses by case/control status using Student’s *t*-test (continuous variables) and chi-square tests (categorical variables) using SAS v9.4. [24]. By this method, significant features at the 0.10-level were included in a logistic regression model (see below). We then began eliminating each covariate from the full model in a stepwise backward fashion, keeping our exposure of interest (*SGCD* SNPs assuming a genotypic mode of inheritance) as a function of AMD diagnosis (yes versus no, comparison 1) or AMD phenotype (GA versus CNV, comparison 2). At each step, we assessed for changes in the remaining coefficients of 10% and compared the −2 likelihood ratios of the full and reduced models to a chi-square distribution, to further determine the significance of each polychotomous independent variable to the overall model. Finally, to produce the most parsimonious model, we re-assessed each eliminated predictor individually in the most reduced model, following the same process described above. Our final model is shown in Tables 3 and 4.

For our genotyping data, we first calculated allelic frequencies for the full sample and stratified by case/control status. We then corroborated them if any of our SNPs significantly deviated from the Hardy–Weinberg equilibrium (HWE), especially in controls, using PLINK v1.90b3.42. [23]. We analyzed only those SNPs whose minor allele frequencies (MAF) were ≥0.01, with HWE ≥0.05 among controls, and a genotyping call rate ≥95% by sequencing and RT-PCR. To decrease any information bias, we also performed all our genotyping assays in duplicate for both conventional sequencing methods and RT-PCR. Also, we estimated haplotypes, haplotype frequencies, and their unadjusted effect on AMD diagnosis using R v3.3.1. [22]. Those haplotypes significant at the 0.10-level were included in a multivariate logistic regression model (see below).

To ascertain the effect of baseline characteristics and genotypes on AMD diagnosis and phenotype (GA versus CNV), we performed unadjusted and adjusted logistic regression models (see above). To simplify our approach, we a priori screened for modes of inheritance using PLINK v1.90b3.42. [23], and discarded those modes of inheritance (e.g., allelic, dominant, recessive, over-dominant) which were not statistically significant. Hence, all our inferential analyses assume a genotypic effect. Our modeling was as follows:

$$log\left(OR_{AMD}\right)∼\mathrm{Clinical}\;\mathrm{features}+\mathrm{SNP}\left(\begin{array}{c} AA_{00}\\ Aa_{01}\\ aa_{10} \end{array}\right)+\in$$

We then selected those genotypes with a significant association at the 0.05 level and included them in a final multivariate logistic regression model. This model was also adjusted by those clinical features significantly associated with either the case/control status (see above) or those significant characteristics in our unadjusted models. To bolster our approach, we included *SGCD* haplotypes (computed with the genetics package in R) of these models using PLINK v1.90b3.42. [23]. Briefly, we took the haplotype objects from R and inputted them into PLINK to calculate the haplotype frequencies in the full sample and stratified them by case/control status. All our modeling was done in PLINK v1.90b3.42. [23] and corroborated in SAS v9.4. [24]. Finally, all of our models were controlled for the familywise error rate using SAS and R, following the Benjamini-Hochberg procedure assuming a standard false discovery rate of 10%. We highlighted in bold all those significant corrected *p*-values.

## 3. Results

### 3.1. Characteristics of the Study Population and Genotyped Data

We studied 268 individuals; half of these were cases diagnosed with AMD. Our sample description is detailed in Table 1. On average, our study population was 74.3 years of age (± 8.2), and 60% or more were at least 70 years or older and female. Almost half had hypertension (HTN), and 27.4% type 2 diabetes mellitus (T2DM). Up to 80% of our sample was either former or current smokers. Among AMD cases, the most prevalent phenotype was GA (69.7%), followed by CNV (30.3%). In our sample, we did not observe any progressions between phenotypes, i.e., GA to CNV. To test if any of these characteristics were significantly associated with the case/control status, we performed stratified analyses, portrayed in Supplementary Table S2.

Controls were significantly five years younger than cases (mean difference of 4.8 years, *p* < 0.0001). Almost half of our cases were 75 years or older compared to controls (70 years or older, *p* < 0.0001). Although this difference is statistically significant, at the clinical level it is expected to have minimal bias, since subjects without any sign of AMD at the age of 70 (as our controls) are extremely improbable to develop the disease [26]. Moreover, obtaining controls without serious ocular alterations affecting retina (AMD, glaucoma, diabetic retinopathy) at an age of 70 years on average is extremely difficult, particularly in hospital-based settings [26]. There were no significant differences in sex, baseline comorbidities (T2DM, HTN), or smoking history between cases and controls. Regarding our genotype data, all four least-frequent alleles in the *SGCD* gene had a minor allele frequency greater than 0.01 (Table 2). Such alleles in our sample are concurrent with already reported SNPs for the Mexican population in Hap Map [27]. Moreover, to provide evidence of bias in our genotyping, we stratified our genotyped SNPs based on their case/control status and tested the HWE, so that significant deviations (*p* < 0.05 χ^2^ test distributed) would indicate a selection bias. All our data followed the HWE-expected distribution without any significant deviations. Furthermore, all of our experiments had a genotyping call rate greater than 95%.

### 3.2. Unadjusted Effect of Baseline Characteristics and Genotypes with Age-Related Macular Degeneration Status and Phenotypes

To test if any characteristic was significantly associated with AMD status, we modeled each feature (either baseline demographics, risk factors, or genotype data) using logistic regression. Our results are portrayed in Supplementary Table S3. We assumed a genotypic mode of inheritance for our genetic data. In our sample, the unadjusted effect of a unit-increase in age is 8% increased odds of AMD (1.08; 95% CI 1.05–1.12; *p* < 0.001). We fail to evidence any significant association with sex, comorbidities, and smoking history. However, the effect of a heterozygote G/A in the rs931798 SNP compared to the G/G ancestral homozygote is 74% increased odds of AMD (95% CI 1.04–2.90, *p* = 0.034). To complement our approach, we then stratified our analyses by AMD phenotype, being either GA or CNV. These results are displayed in Supplementary Table S4. Age is significantly associated with both phenotypes (Supplementary Table S4). For each case, a unit increase elevates the odds of being diagnosed with GA in 8% and CNV in 9% of cases (*p* < 0.0001 for both comparisons). Furthermore, the bivariate effect of the above G/A genotype seems to be associated with the geographic atrophy phenotype, as that increases the odds of GA by 82% compared to the G/G genotype (95% CI 1.03–3.21, *p* = 0.038).

### 3.3. Adjusted Effect of Baseline Characteristics and Genotypes with Age-related Macular Degeneration Status

To determine the independent effect of our significant predictors in the unadjusted analyses, we performed a multivariable logistic regression model of those features significantly associated with AMD or those with clinical significance. Our results are displayed in Table 3. After adjusting for age and sex in our sample, the independent effect of the G/A genotype in the rs931798 SNP is 81% increased odds of AMD compared to the G/G ancestral genotype (95% CI 1.06–3.14, *p* = 0.031). Also, a one-unit increase in age increases the odds of AMD by 8% (95% CI 1.05–1.12, *p* < 0.0001). To further corroborate the effects on the AMD phenotype (see above), we additionally ran controlled stratified analyses portrayed in Table 4. The independent effect of the G/A genotype is seen in the geographic atrophy phenotype. Compared to the G/G genotype, the G/A heterozygous is associated with 81% increased odds of geographic atrophy, holding constant sex and age (1.01–3.26, *p* = 0.038). Age is significantly associated with both GA and CNV. Holding constant all variables in the model, a unit-increase in age increased both the odds of GA and the odds of CNV by 8% (Table 4).

### 3.4. Haplotype Analyses

To bolster our approach, we also constructed haplotype configurations and regressed in a logistic genotypic model. The haplotype GATT (rs931798, rs140617, rs140616, and rs970476) (Supplementary Table S5) is associated with 86% decreased odds of being diagnosed with AMD (95% CI 0.02–0.93, *p* = 0.011). However, it is less frequent in our population, and its coefficient of linkage disequilibrium (D′) is 52 (Supplementary Figure S1). After adjusting for other covariates in a multivariate regression model, the independent effect of the GATT haplotype remained (Table 5). This haplotype significantly decreases the odds of being diagnosed with AMD in 87% of cases (95% CI 0.02–0.91, *p* = 0.41). However, we were not able to produce stratified analyses by phenotype given our sample size. There were more statistically significant haplotype configurations (Supplementary Figure S1) based on D′ values; however, these three SNP configurations were not statistically significant in a logistic regression model of AMD diagnosis.

## 4. Discussion

The global burden of age-related macular degeneration on severe visual impairment and legal blindness is increasing. For North America, in 2015, around 15.9% of regional blindness was directly attributable to AMD in those aged 50 years or older [3]. Moreover, there is a disproportionate prevalence of cases among high-income countries, especially those whose population is aging. Since this disease is multifactorial, there is a great interest in characterizing molecular underpinnings and environmental exposures that may influence AMD phenotype, prognosis, progression, and response to treatment, with the aim of decreasing AMD incidence [28]. Herein, we provide experimental evidence of a novel association between the *SGCD* gene and AMD diagnosis and phenotype.

δ-sarcoglycan is a structural protein that forms a complex with dystrophin among others [29]. The gene product—δ-SG—is expressed diffusely in the murine retina, but in abundance around the external (photoreceptors, external limiting membrane ELM) and internal (internal limiting membrane ILM, optic nerve, ganglion cells) segments [20]. The significance or role of the retinal function or homeostasis is poorly understood. Key partners, such as dystrophin-71 (Dp71) expressed in Müller glial cells, aid in regulating the extracellular milieu and potassium homeostasis [30]. Others, such as the sarcoglycan complex, promote intercellular junctions and thereby stability [20]. We have also evidenced that *SGCD*-null mice had increased retinal frailty compared to a wild-type, as well as decreased layer thickness, and electroretinogram aberrances [21]. These results strongly support the relevance of δ-sarcoglycan and its partners and encourage future characterization of this protein in the development of retinal impairment, a trait shared in many retinal diseases.

On top of these promising results in animal studies, others have evidenced an association of *SGCD* with AMD in silico. Tang et al. provided intriguing results of biomarkers in this gene interacting with the SCAPER gene [15]. Even further, in a secondary prioritized subset analyses from already well-established AMD cohorts, Lin identified several SNPs in silico that might increase the odds of AMD [16]. Here, we examined these markers in a case-control study done with Mexicans. The G/A genotype from rs931798 increased the odds for AMD, specifically for the geographic atrophy phenotype. Moreover, there might be an encouraging haplotype of all these biomarkers, but future studies warrant its replication. Currently, the specific function of this SNP is unknown. A plausible role might be in modulating complement levels in drusen [15]. In support of this hypothesis are co-expression analyses that correlate both *SGCD* and *SCAPER* with *MASP1* (*p*-value, < 1 × 10^−5^) and *MASP2* (*p*-value, < 1 × 10^−2^) genes. Both are activators of the complement pathway [15]. Also, it may exert influence through the regulation of the aging process, as pieces of evidence show that *SCAPER-SGCD* epistasis might be involved in cell cycle regulation and DNA repair. Moreover, all of these four SNPs we have evaluated are in significant linkage disequilibrium (r^2^ > 0.8) with a neighboring region significantly associated with the expression of *FBLN1* [15]. Members of this family have been associated with AMD, as they can act as cofactors in silico for the matrix metalloprotease *ADAMTS1*, and may play a role in proteoglycan degradation by *ADAMTS1* under inflammation [15]. Biomarkers in *SGCD* might also favor the development of AMD, especially of the geographic atrophy phenotype, in a similar pathway to *HTRA1*, by regulating the degradation of the extracellular matrix and facilitating access of other degradative matrix enzymes like matrix metalloproteinases to their substrates [15].

Our findings, though promising, are exploratory, and limited to the Mexican population. Despite our sample size, we were powered to detect significant differences in odds from 1.2 and higher. Future replication in other ethnicities is warranted to test the effect of such biomarkers on disease phenotype and progression. A new line of research should focus on dissecting the molecular pathway of *SGCD* in AMD, especially characterizing retinal alterations in patients with Limb-Girdle Muscle Dystrophies caused by disruptions of this gene.

## Figures and Tables

**Table 1:** Description of the sample (*n* = 268).

Characteristic	*n* (%) *
Age (years), mean ± SD	74.3 ± 8.2
Age (years), *n* (%)	
(50–60)	7 (2.6)
(60–65)	23 (8.6)
(65–70)	46 (17.2)
(70–75)	60 (22.5)
(75–80)	59 (22.1)
≥80	72 (27.0)
Sex	
Male	97 (36.2)
Female	171 (63.8)
Type 2 diabetes, *n* (%)	71 (27.4)
Hypertension, *n* (%)	134 (51.0)
Smoking history, *n* (%)	
Never	202 (80.5)
Former or current	49 (19.5)
AMD phenotype, *n* (%) ^	
Geographic atrophy (GA)	92 (69.7)
Neovascular (NV)	40 (30.3)

Full sample description. Means and standard deviations, along with sample size and column percentages are shown. We excluded one individual because of being younger than 50 years old. * Numbers may not sum to totals due to missing data, and column percentages may not sum to 100% due to rounding. ^ Among cases (*n* = 134).

**Table 2:** Minor allele frequencies and the Hardy–Weinberg equilibrium (*n* = 268).

SNP	A1 *	A2	Pooled (*n* = 268)	Cases (*n* = 134)	Controls (*n* = 134)	*p* ^†^	MAF Mex	MAF C	MAF AfAm	MAF A
			MAF	MAF	MAF					
rs970476	T	G	0.455	0.459	0.452	0.601	–	0.458	0.750	0.167
rs931798	A	G	0.330	0.358	0.302	0.538	0.280	0.283	0.010	0.167
rs140617	G	A	0.164	0.160	0.168	0.766	0.170	0.102	0.117	0.220
rs140616	C	T	0.494	0.485	0.504	1.000	0.480	0.575	0.295	0.756

Genetic data summary with allele frequencies (A1, A2) of the full sample (pooled), and stratified by case/control status. The least frequent allele in our sample (A1) follows those frequencies of single nucleotide polymorphisms (SNPs) in the Mexican population (MAF Mex) reported in HapMap [27]. The Hardy-Weinberg equilibrium (HWE) χ^2^ test is shown to indicate any significant deviations, especially in controls. Shaded in gray as a comparison are minor allele frequencies in HapMap [27] among Mexicans (Mex), Caucasians (C), African Americans (AfAM), and Asians (A). † The *p*-value for HWE χ^2^ test evaluated at the 0.05 level. * All least frequent alleles for our sample. – No data available. CHR: chromosome; SNP: rs ID; A1: least frequent allele in the sample; MAF: minor allele frequency; *p*: significance of HWE in controls.

**Table 3:** Multivariable logistic regression model of factors associated with age-related macular degeneration (AMD) (*n* = 268).

Characteristic	Adjusted Odds Ratio (OR (95% CI))	*p* ^†^
Age (years)	1.08 (1.05, 1.12)	<0.0001
Sex		
Female	1.00	–––
Male	0.63 (0.37, 1.08)	0.096
rs931798		
G/G	1.00	–––
**G/A**	**1.81 (1.06, 3.14)**	**0.031**
A/A	1.11 (0.45, 2.69)	0.822

Adjusted associations between baseline characteristics and AMD diagnosis (0–No, 1–Yes AMD). For genetic data, we assumed a genotypic mode of inheritance. Such models follow $\log\left(OR_{AMD}\right)∼\mathrm{Baseline}\;\mathrm{characteristics}+\;\mathrm{SNP}\left(\begin{array}{c} AA_{00}\\ Aa_{01}\\ aa_{10} \end{array}\right)+\in$, where *AA* is the most frequent allele in our population, taken as reference. We considered statistically significant predictors of odds of disease those whose *p*-value < 0.05 held constant for all variables in the model. † The *p*-value for β significance adjusted for other covariates. In bold are significant predictors at the 0.05 level. – Level set as reference.

**Table 4:** Multinomial logistic regression model of factors associated with the AMD phenotype (*n* = 268).

Characteristic *	Geographic Atrophy Odds Ratio (OR (95% CI))	*p* ^†^	Neovascular Odds Ratio (OR (95% CI))	*p* ^†^
SNP2 ^^^				
G/G	1.00	–––	1.00	–––
**G/A**	**1.81 (1.01, 3.26)**	**0.047**	**1.40 (0.65, 3.01)**	**0.393**
A/A	1.24 (0.47, 3.24)	0.666	1.09 (0.31, 3.87)	0.890
**Age(years)**	**1.08 (1.04, 1.12)**	**<0.0001**	**1.09 (1.04, 1.15)**	**<0.0001**

Adjusted associations between baseline characteristics and AMD phenotype (either 1–GA, 0–else; or 1–NV, 0–else). For genetic data, we assumed a genotypic mode of inheritance. Such models follow: $\log\left(OR_{AMD}\right)∼\mathrm{Baseline}\;\mathrm{characteristics}+\;\mathrm{SNP}\left(\begin{array}{c} AA_{00}\\ Aa_{01}\\ aa_{10} \end{array}\right)+\in$, where *AA* is the most frequent allele in our population, taken as reference. We considered statistically significant predictors of odds of disease those whose *p*-value < 0.05 held constant for all variables in the model. † The *p*-value for β significance. NS: not significant at the 0.05 level. * Controls or non-diseased phenotype are set as reference for all multinomial logistic regression models. ^ We took the most common allele and set it as reference. In bold significant predictors at the 0.05 level. – Level set as reference.

**Table 5:** Multivariable logistic regression model of haplotypes and factors associated with AMD (*n* = 268).

Characteristic	Adjusted OR (95% CI)	*p* ^†^
Age (years)	**1.09 (1.05, 1.13)**	**<0.0001**
Haplotype #1 (GATT)	**0.13 (0.02, 0.91)**	**0.041**

Adjusted associations between haplotype configurations of SNPs in the SGCD gene and AMD diagnosis by logistic regression. † The *p*-value for β significance. In bold are significant predictors at the 0.05 level.

## Supplementary Materials

The following are available online at http://www.mdpi.com/2073-4425/9/10/467/s1. Figure S1: Heat map and D´ values of SGCD single nucleotide polymorphisms, Table S1: Case and control definition, Table S2: Description of the sample by case/control status (n = 268), Table S3: Unadjusted associations between study variables and age-related macular degeneration (n = 268), Table S4: Unadjusted associations between study variables and age-related macular degeneration phenotype (n = 268), Table S5: Unadjusted haplotypes of four SNPs with AMD (n = 268).

## References

[1] Wong W.L., Su X., Li X., Cheung C.M.G., Klein R., Cheng C.Y., Wong T.Y. (2014). Global prevalence of age-related macular degeneration and disease burden projection for 2020 and 2040: A systematic review and meta-analysis. Lancet Glob. Health.

[2] Pascolini D., Mariotti S.P. (2012). Global estimates of visual impairment: 2010. Br. J. Ophthalmol..

[3] Bourne R.R.A., Flaxman S.R., Braithwaite T., Cicinelli M.V., Das A., Jonas J.B., Keeffe J., Kempen J.H., Leasher J., Limburg H. (2017). Magnitude, temporal trends, and projections of the global prevalence of blindness and distance and near vision impairment: A systematic review and meta-analysis. Lancet Glob. Health.

[4] Zhang K., Zhang L., Weinreb R.N. (2012). Ophthalmic drug discovery: Novel targets and mechanisms for retinal diseases and glaucoma. Nat. Rev. Drug Discov..

[5] Rattner A., Nathans J. (2006). Macular degeneration: Recent advances and therapeutic opportunities. Nat. Rev. Neurosci..

[6] Crabb J.W. (2014). The proteomics of drusen. Cold Spring Harb. Perspect. Med..

[7] Lim L.S., Mitchell P., Seddon J.M., Holz F.G., Wong T.Y. (2012). Series ophthalmology 1 age-related macular degeneration. Lancet.

[8] Chakravarthy U., Wong T.Y., Fletcher A., Piault E., Evans C., Zlateva G., Buggage R., Pleil A., Mitchell P. (2010). Clinical risk factors for age-related macular degeneration: A systematic review and meta-analysis. BMC Ophthalmol..

[9] Haines J.L. (2005). Complement factor H variant increases the risk of age-related macular degeneration. Science.

[10] Sardell R.J., Persad P.J., Pan S.S., Whitehead P., Adams L.D., Laux A., Fortun J.A., Bartnley M.A., Kovach J.L., Schwartz S.G. (2016). Progression rate from intermediate to advanced age- related macular degeneration is correlated with the number of risk alleles at the CFH locus. Invest. Ophthalmol. Vis. Sci..

[11] Geerlings M.J., de Jong E.K., den Hollander A.I. (2017). The complement system in age-related macular degeneration: A review of rare genetic variants and implications for personalized treatment. Mol. Immunol..

[12] Sivakumaran T.A., Igo R.P., Kidd J.M., Itsara A., Kopplin L.J., Chen W., Hagstrom S.A., Peachey N.S., Francis P.J., Klein M.L. (2011). A 32 kb critical region excluding Y402H in CFH mediates risk for age-related macular degeneration. PLoS ONE.

[13] Pennington K.L., DeAngelis M.M. (2016). Epidemiology of age-related macular degeneration (AMD): Associations with cardiovascular disease phenotypes and lipid factors. Eye Vis..

[14] Fritsche L.G., Chen W., Schu M., Yaspan B.L., Yu Y., Thorleifsson G., Zack D.J., Arakawa S., Cipriani V., Ripke S. (2013). Seven new loci associated with age-related macular degeneration. Nat. Genet..

[15] Tang W., Wu X., Jiang R., Li Y. (2009). Epistatic module detection for case-control studies: A Bayesian model with a Gibbs sampling strategy. PLoS Genet..

[16] Lin W.-Y., Lee W.-C. (2010). Incorporating prior knowledge to facilitate discoveries in a genome-wide association study on age-related macular degeneration. BMC Res. Notes.

[17] Hashimoto R., Yamaguchi M. (2006). Genetic link between b—Sarcoglycan and the Egfr signaling pathway. Biochem. Biophys. Res. Commun..

[18] Tsubata S., Bowles K.R., Vatta M., Zintz C., Titus J., Muhonen L., Bowles N.E., Towbin J.A. (2000). Mutations in the human delta-sarcoglycan gene in familial and sporadic dilated cardiomyopathy. J. Clin. Invest..

[19] Costa M.F., Oliveira A.G.F., Feitosa-Santana C., Zatz M., Ventura D.F. (2007). Red-green color vision impairment in Duchenne muscular dystrophy. Am. J. Hum. Genet..

[20] Fort P., Estrada F.J., Bordais A., Mornet D., Sahel J.A., Picaud S., Vargas H.R., Coral-Vázquez R.M., Rendon A. (2005). The sarcoglycan-sarcospan complex localization in mouse retina is independent from dystrophins. Neurosci. Res..

[21] Perez-Ortiz A.C., Solano-García G., Luna-Angulo A., Coral-Vazquez R.M., Garfias Y., Garcia-Perez V., De los Santos-Enriquez S., Rendon A., Ramirez-Sanchez I., Estrada-Mena F.J. (2016). Changes in the sarcoglycan complex and effects of (-)-epicatechin in SGCD-null mice as a potential animal model for retinal degeneration. Invest. Ophthalmol. Vis. Sci..

[22] Developement Core Team R (2015). A language and environment for statistical computing. R Found. Stat. Comput..

[23] Purcell S., Neale B., Todd-Brown K., Thomas L., Ferreira M.A.R., Bender D., Maller J., Sklar P., de Bakker P.I.W., Daly M.J. (2007). PLINK: A tool set for whole-genome association and population-based linkage analyses. Am. J. Hum. Genet..

[24] (2014). SAS software 9.4.

[25] American Academy of Ophthalmology—Retina/Vitreous Panel (2015). Age-Related Macular Degeneration.

[26] Hernández-Narváez M.G., Olivares-Luna A.M., Carillo-Hernández A., Tovar-Méndez G.M., González-Pedraza Avilés A. (2015). Prevalencia de trastornos visuales y su relación con la funcionalidad en adultos mayores Prevalence of visual disorders and their relationship with functionality of the older adults. Rev. Cuba. Oftalmol..

[27] The International HapMap Consortium (2003). The International HapMap project. Nature.

[28] Ryu E., Fridley B.L., Tosakulwong N., Bailey K.R., Edwards A.O. (2010). Genome-wide association analyses of genetic, phenotypic, and environmental risks in the age-related eye disease study. Mol. Vis..

[29] Blake D.J., Weir A., Newey S.E., Davies K.E. (2002). Function and genetics of dystrophin and dystrophin-related proteins in muscle. Physiol. Rev..

[30] Claudepierre T., Dalloz C., Mornet D., Matsumura K., Sahel J., Rendon A. (2000). Characterization of the intermolecular associations of the dystrophin-associated glycoprotein complex in retinal Müller glial cells. J. Cell. Sci..

