# Supplementary material for: Significant Association Between Variant in SGCD and Age-Related Macular Degeneration
Source: Genes (Basel). 2018 Sep 25;9(10):467. doi: 10.3390/genes9100467 (PMC6210939; doi:10.3390/genes9100467)
Supplement: Supplementary file 1 [file genes-09-00467-s001.pdf]

SUPPLEMENTARY TABLES

Table 1. Case and control definition.

AMD Cases (n = 134)	Hospital-based controls (n = 134)
65 years or older.<br>Any gender.<br>With or without a family history of AMD.<br>AMD CARMS grades 4 or 5†.<br>No history of vitreoretinal procedures.<br>No other concurrent retinal diseases.	65 years or older.<br>Any gender.<br>No family history of AMD.<br>No retinal changes suggestive of advanced AMD by fundoscopy.<br>Drusen less than 65 μm in diameter by fundoscopy. #

The presence of a few small hard drusen less than 65 μm in diameter is common and is no longer considered to be a risk factor for the development of age related maculopathy[31]

Table 2. Description of the sample by case/control status (n = 268).

Characteristic	Cases (n = 134)*	Controls (n = 134)*	p†
Age (years), mean ± SD	**76.7 ± 7.6**	**71.9 ± 8.1**	**<0.0001**
Age (years), n (%)			**<0.0001**
[50 – 60)	**0 (0.0)**	**7 (5.3)**	
[60 – 65)	**7 (5.2)**	**16 (12.0)**	
[65 – 70)	**18 (13.4)**	**28 (21.1)**	
[70 – 75)	**28 (20.9)**	**32 (24.1)**	
[75 – 80)	**30 (22.4)**	**29 (21.8)**	
≥ 80	**51 (38.1)**	**21 (15.8)**	
Sex, n (%)			0.098
Male	42 (31.3)	55 (41.0)	
Female	92 (68.7)	79 (59.0)	
Type 2 diabetes, n (%)	32 (24.4)	39 (30.5)	0.276
Hypertension, n (%)	73 (54.5)	61 (47.3)	0.244
Smoking history, n (%)			0.741
Never	106 (79.7)	96 (81.4)	
Former or current	27 (20.3)	22 (18.6)	

Stratified characteristics by case/control status. Statistically significant differences are shown in bold and were computed with Student's t-Test (Continuous variables) or χ^2 test (categorical variable). We excluded one subject from this analysis because of age (<50 years old).

* Numbers may not sum to totals due to missing data, and column percentages may not sum to 100% due to rounding.

† P-value for Student's t-test (continuous variable) or χ^2 test (categorical variable).

^ One subject was excluded because of being younger than 50 years old.

In bold significant characteristics at the 0.05 level.

Table 3. Unadjusted associations between study variables and age-related macular degeneration (n = 268).

Characteristic^	N* (% with AMD)	OR (95% CI)	p†
rs970476			
G/G	41 (30.6)	1.00	—
G/T	63 (47.0)	1.02 (0.59, 1.79)	NS
T/T	30 (22.4)	1.06 (0.54, 2.07)	NS
rs931798			
G/G	51 (38.1)	1.00	—
**G/A**	**70 (52.2)**	**1.74 (1.04, 2.90)**	**0.034**
A/A	13 (9.7)	1.22 (0.52, 2.83)	NS
rs140617			
A/A	97 (72.4)	1.00	—
G/A	31 (23.1)	0.80 (0.46, 1.40)	NS
G/G	6 (4.5)	1.42 (0.40, 5.78)	NS
rs140616			
T/T	34 (25.4)	1.00	—
T/C	70 (52.2)	1.01 (0.56, 1.82)	NS
C/C	30 (22.4)	0.86 (0.43, 1.70)	NS
**Age(years)**	**–**	**1.08 (1.05, 1.12)**	**<0.0001**
Sex			
Female	92 (68.7)	1.00	—
Male	42 (31.3)	0.66 (0.40, 1.08)	NS
Type 2 diabetes			
No	99 (75.6)	1.00	—
Yes	32 (24.4)	0.73 (0.42, 1.28)	NS
Hypertension			
No	61 (45.5)	1.00	—
Yes	73 (54.5)	1.33 (0.82, 2.17)	NS
Smoking history			
Never	106 (79.7)	1.00	—
Former or current	27 (20.3)	1.11 (0.60, 2.08)	NS

Bivariate associations between baseline characteristics and AMD diagnosis (0–No, 1–Yes AMD). For genetic data, we assumed a genotypic mode of inheritance. Such models follow: $\log(OR_{AMD}) \sim \text{SNP}\begin{pmatrix} AA_{00} \\ Aa_{01} \\ aa_{10} \end{pmatrix} + \in$ where: AA is the most frequent allele in our population, taken as reference. We considered statistically significant predictors of odds of disease those whose p-value < 0.05.

* Numbers may not sum to total due to missing data.

† p-value for β significance

NS: not significant at the 0.05 level.

^We took the most common allele for each case and set it as reference. Effects displayed first as those of the intercept for each model.

~ One subject was excluded because of being younger than 50 years old.

In bold significant predictors at the 0.05 level.

Table 4. Unadjusted associations between study variables and age-related macular degeneration phenotype (n = 268).

Characteristic*	Geographic atrophy OR (95% CI)	p†	Neovascular OR (95% CI)	p†
rs931798^				
G/G	1.00	—	1.00	—
**G/A**	**1.82 (1.03, 3.21)**	**0.038**	1.41 (0.67, 2.98)	NS
A/A	1.27 (0.50, 3.22)	NS	1.13 (0.33, 3.86)	NS
**Age(years)**	**1.08 (1.04, 1.12)**	**<0.0001**	**1.09 (1.04, 1.14)**	**<0.0001**
Sex				
Female	1.00	—	1.00	—
Male	0.70 (0.40, 1.21)	NS	0.54 (0.25, 1.18)	NS
Type 2 diabetes				
No	1.00	—	1.00	—
Yes	0.65 (0.35, 1.22)	NS	0.86(0.39, 1.92)	NS
Hypertension				
No	1.00	—	1.00	—
Yes	1.39 (0.81, 2.38)	NS	1.23 (0.61, 2.5	NS
Smoking history				
Never	1.00	—	1.00	—
Former or current	1.32 (0.67, 2.56)	NS	0.77 (0.29, 2.04)	NS

Bivariate associations between baseline characteristics and AMD phenotype (either 1–GA, 0–else; or 1–NV, 0–else). For genetic data, we assumed a genotypic mode of inheritance. Such models follow: $\log(OR_{AMD\ phenotype}) \sim \text{SNP}\begin{pmatrix} AA_{00} \\ Aa_{01} \\ aa_{10} \end{pmatrix} + \in$ where: AA is the most frequent allele in our population, taken as reference. We considered statistically significant predictors of odds of disease those whose p-value < 0.05.

† p-value for β significance

NS: not significant at the 0.05 level.

*Controls or non-diseased phenotype are set as reference for all multinomial logistic regression models.

^We took the most common allele and set it as reference.

In bold significant predictors at the 0.05 level.

Table 5. Unadjusted haplotypes of four SNPs with AMD (n = 268).

#	SNP 1	SNP 2	SNP 3	SNP 4	Pooled HF	Control HF	Case HF	OR (95% CI)	p†
**1**	**G**	**A**	**T**	**T**	**0.022**	**0.039**	**0.006**	**0.14 (0.02, 0.93)**	**0.011**
2	A	G	T	T	0.012	0.019	0.005	0.21 (0.02, 2.04)	0.216
3	G	A	C	T	0.099	0.107	0.089	0.82 (0.44, 1.51)	0.428
4	A	A	T	G	0.017	0.022	0.012	0.62 (0.16, 2.41)	0.530
5	G	A	T	G	0.022	0.022	0.021	1.00 (0.27, 3.75)	0.754
6	G	A	C	G	0.378	0.384	0.375	1.00 (NA, NA)	0.896
7	G	G	T	G	0.114	0.114	0.115	0.97 (0.52, 1.81)	0.956
8	G	G	T	T	0.025	0.023	0.027	1.84 (0.42, 7.98)	0.857
9	A	A	T	T	0.293	0.257	0.329	1.31 (0.84, 2.02)	0.084

Haplotype configurations of SNPs in the SGCD gene. We show their frequency (HF) in the full sample (Pooled HF) and stratified by case/control status. Also, bivariate associations between a haplotype configuration (#) and AMD diagnosis using logistic regression modeling.

SNP1: rs931798, SNP2: rs140617, SNP3: rs140616, SNP4: rs970476.
# Four single-nucleotide polymorphism (SNP) haplotype configuration.
† p-value for haplotype $\chi 2$ test evaluated at the 0.05 level.
HF: Haplotype frequency among cases, controls, and full study sample (pooled).
NA: Not able to calculate by this method.
In bold significant haplotypes at the 0.05 level.
